# The relationship between remnant cholesterol and the risk of testosterone deficiency in US adults: a cross-sectional study based on the NHANES database

**DOI:** 10.3389/fendo.2024.1458193

**Published:** 2024-09-24

**Authors:** Yangyang Mei, Yiming Chen, Xiaogang Wang, Renfang Xu, Xingliang Feng

**Affiliations:** ^1^ Department of Urology, Jiangyin People's Hospital Affiliated to Nantong University, Wuxi, Jiangsu, China; ^2^ Department of Urology, The First People’s Hospital of Changzhou, Changzhou, Jiangsu, China; ^3^ Department of Urology, The Third Affiliated Hospital of Soochow University, Changzhou, Jiangsu, China

**Keywords:** testosterone deficiency, remnant cholesterol, NHANES, men’s health, cross-sectional study

## Abstract

**Background:**

Testosterone deficiency (TD) is an urgent health issue that requires attention, associated with various adverse health outcomes including cardiovascular diseases (CVD) and metabolic syndrome. Remnant cholesterol (RC) has emerged as a potential biomarker for cardiovascular risk, but its relationship with testosterone levels and TD has not been thoroughly investigated. This study aims to explore the association between RC and TD in adult American males using data from the National Health and Nutrition Examination Survey (NHANES).

**Methods:**

This cross-sectional study utilized data from three NHANES cycles (2011-2016), including 2,848 adult male participants. RC was calculated as total cholesterol minus high-density lipoprotein cholesterol (HDL) and low-density lipoprotein cholesterol (LDL). TD was defined as total testosterone levels below 300 ng/dL. Multivariable linear and logistic regression analyses, as well as smooth curve fitting and generalized additive models, were performed to assess the associations between RC and total testosterone levels and TD, adjusting for potential confounders. Subgroup analyses were conducted based on age, BMI, smoking status, diabetes, hypertension, CVD, and chronic kidney disease (CKD).

**Results:**

Higher RC levels were significantly associated with lower total testosterone levels (β = -53.87, 95% CI: -77.69 to -30.06, p<0.001) and an increased risk of TD (OR = 1.85, 95% CI: 1.29 to 2.66, p=0.002) in fully adjusted models. When RC was analyzed as quartiles, participants in the highest quartile (Q4) had significantly lower total testosterone levels (β = -62.19, 95% CI: -93.62 to -30.76, p<0.001) and higher odds of TD (OR = 2.15, 95% CI: 1.21 to 3.84, p=0.01) compared to those in the lowest quartile (Q1). Subgroup analyses revealed consistent associations across different age groups, particularly strong in participants over 60 years, and in never smokers. The associations remained significant in both hypertensive and non-hypertensive groups, as well as in those with and without CKD. No significant interactions were found across subgroups.

**Conclusion:**

This study demonstrates a significant inverse association between RC levels and total testosterone levels, along with a positive association with the risk of TD. These findings suggest that RC could serve as a valuable biomarker for early identification of individuals at risk for TD. Future longitudinal studies are needed to confirm these findings and explore the underlying mechanisms.

## 1 Introduction

Testosterone is the primary male sex hormone, predominantly produced by the Leydig cells in the testes, with minor contributions from the adrenal glands (1). In men, testosterone secretion peaks during adolescence and early adulthood, then gradually declines at a rate of 0.4-2.6% per year after the age of 40, potentially culminating in a reduction of 20% to 50% by the age of 80 (2, 3). The testosterone, instrumental in promoting reproductive and sexual functions, is equally crucial for maintaining cardiovascular health, cognitive function, and bone integrity, orchestrating a symphony of biological processes essential to men’s health (4–7). Consequently, a decline or deficiency in testosterone levels naturally leads to a spectrum of organ dysfunctions, manifesting as decreased semen quality, reduced libido, erectile dysfunction, and an increased risk of cardiovascular diseases (CVD), cognitive decline, depression, and fractures, which collectively constitute testosterone deficiency syndrome (TDs), or hypogonadism (8). Epidemiological data indicate that the risk of testosterone deficiency (TD) in men over the age of 50 ranges from 0.6% to 12%, with an average prevalence of approximately 7%, and this prevalence alarmingly increases with advancing age (9, 10). Given the inexorable global trend of an aging population, the attendant risk of declining testosterone levels is becoming a focal point of concern, necessitating the identification of predictive factors for early detection and timely clinical intervention to mitigate associated health risks.

Many studies have extensively explored the relationship between hyperlipidemia/lipid metabolism disorders and declining testosterone levels. However, the conclusions drawn from these studies have been inconsistent. Some research has reported that high triglycerides (TG) and low high-density lipoprotein (HDL) levels are associated with low testosterone levels (11, 12), whereas other studies have found no significant relationship between high total cholesterol (TC), high low-density lipoprotein (LDL), and testosterone levels (13, 14). These conflicting results have hindered the clinical use of traditional lipid profiles in precisely predicting the risk of declining testosterone levels or TD, making the search for new lipid markers to predict lower testosterone levels even more urgent. In recent years, a new lipid metabolism marker, remnant cholesterol (RC), has gained the attention of researchers (15). RC represents the amount of cholesterol in the remnant lipoproteins transformed from triglyceride-rich lipoproteins in the blood, mainly including chylomicron remnants, very-low-density lipoproteins (VLDL), and intermediate-density lipoproteins (IDL). It is calculated by subtracting the HDL and LDL cholesterol from the TC (16). Numerous cross-sectional and cohort studies have confirmed its close relationship with CVD and metabolic syndrome, which are closely related to risk of TD (17, 18). Therefore, the impact of RC on the risk of TD warrants further exploration, but it remains an unresolved area of research to date.

Given the gaps and inconsistencies in current research, this study aims to investigate the relationship between RC and the risk of TD in adult American males using data from the National Health and Nutrition Examination Survey (NHANES). This nationally representative database, with its large sample size, comprehensive information, and high data quality, allows for detailed and generalizable analysis. We hypothesize that elevated RC levels are associated with an increased risk of TD, potentially serving as a new predictor for TD and thereby informing early identification and intervention strategies for high-risk populations.

## 2 Materials and methods

### 2.1 Study design and study subjects

The NHANES is a national health and nutrition program managed by the National Center for Health Statistics (NCHS) under the Centers for Disease Control and Prevention (CDC). Conducted every two years, NHANES aims to assess the health and nutritional status of adults and children in the United States, providing critical data for health management and policy-making. To ensure the representativeness of the selected sample population, NHANES employs a multistage, complex probability sampling method. All data collection, including demographics, dietary, physical examinations, laboratory tests, and questionnaires, is performed by well-trained experts following standardized procedures to ensure data quality. Ethical approval for NHANES was obtained from the NCHS Research Ethics Review Board, and all participants provided written informed consent prior to participation.

**Figure 1:**
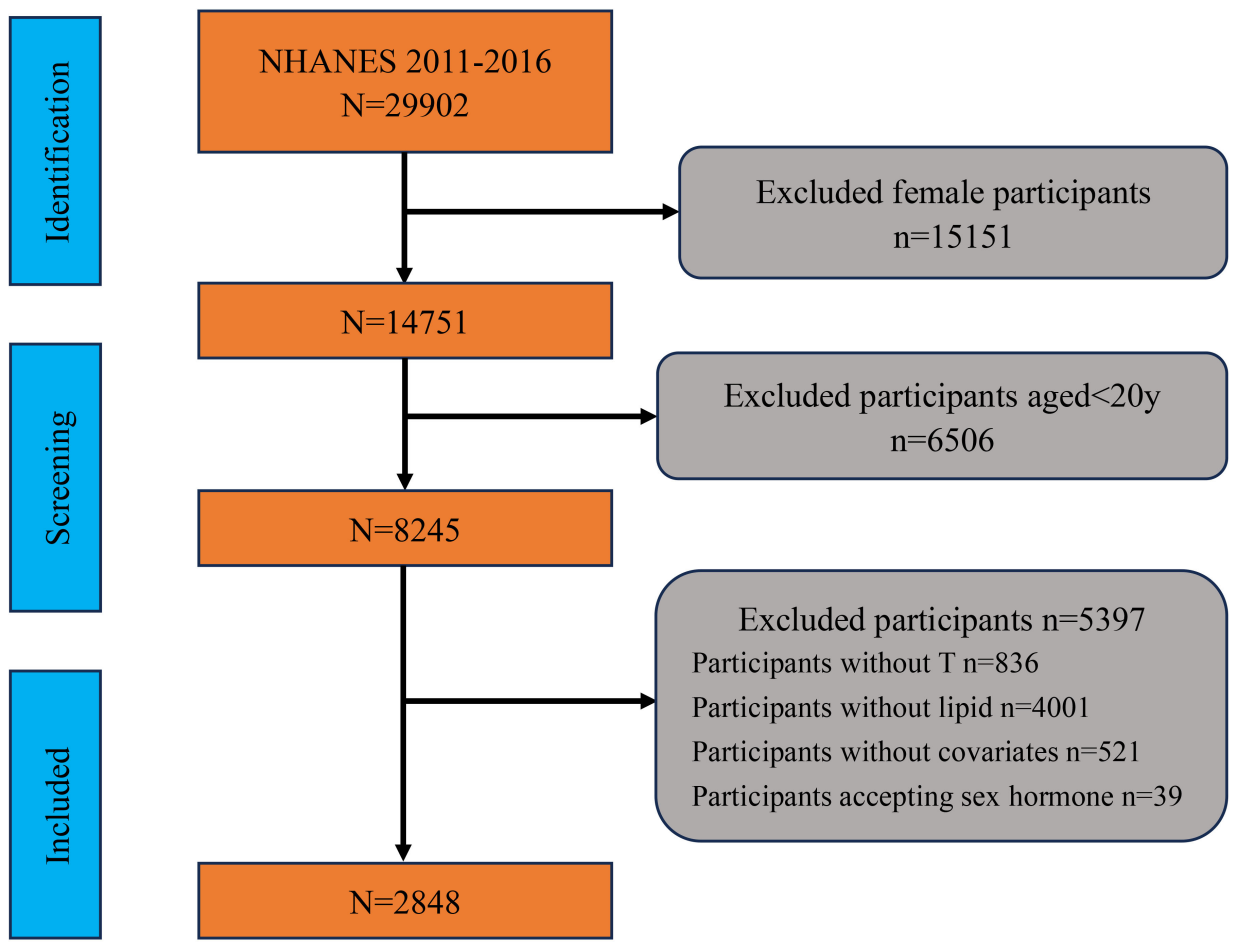
Flowchart of participant selection and exclusion criteria. NHANES, National Health and Nutrition Examination Survey; T, testosterone.

For this study, data from three NHANES cycles (2011-2012, 2013-2014, 2015-2016) were utilized, as these cycles included sexual hormone level assessments. The inclusion criteria were limited to adult males aged 20 years and older with available data on RC and testosterone levels. Initially, the total number of participants in the NHANES dataset was 29,902. We first excluded 15,151 female participants, followed by participants under the age of 20. Next, we excluded 836 participants who did not complete testosterone measurements and 4,001 participants who did not complete lipid measurements. We also excluded 39 participants who were on sex hormone therapy. Finally, we excluded 521 participants who lacked data on covariates. After these exclusions, a total of 2,848 adult male participants with complete data on RC, testosterone levels, and covariates were included in the final analysis. And the detailed sample selection process was shown in **Figure 1**.

### 2.2 Assessment of remnant cholesterol, testosterone levels, and testosterone deficiency in NHANES

All peripheral blood samples were collected in the mobile examination center (MEC) during the morning, with participants required to fast for at least 8 hours. The collected blood samples were properly stored and transported at -20°C to the Lipoprotein Analytical Laboratory at Johns Hopkins University for analysis. Serum TG and TC were measured using the enzymatic method, while serum HDL-C was determined using either the heparin-manganese precipitation method or a direct immunoassay technique. LDL-C was not measured directly; instead, it was calculated using the Friedewald equation, which is [LDL] = [TC] - [HDL] - [TG/5]. According to international guidelines, RC was calculated as RC = TC - HDL- LDL (19).

Testosterone levels were measured using the same sample collection and handling procedures. Based on the National Institute for Standards and Technology’s (NIST) reference method, total testosterone was measured using isotope dilution liquid chromatography-tandem mass spectrometry (ID-LC-MS/MS), with a lower detection limit of 0.75 ng/ml. According to the guidelines of the American Urological Association, TD was defined as a total testosterone level below 300 ng/ml (20).

All sample collection methods and requirements can be found on the NHANES website (www.cdc.gov/nchs/nhanes), and consistent detection methods and quality control standards across different cycles ensured the reliability and combinability of the data.

### 2.3 Potential covariates of interest

The selection of potential covariates in this study was based on previous literature, aiming to include as many factors as possible that might influence the relationship between RC and TD (14, 21). These covariates included demographics, lifestyle behaviors, hematological indicators, and medical comorbidities. Demographic characteristics included age, body mass index (BMI), race/ethnicity, poverty income ratio (PIR) representing household income, education level, and marital status. Participants were categorized into three age groups: 20-40y, 40-60y, and >60y. BMI was classified into three categories: normal (<25 kg/m²), overweight (25-30 kg/m²), and obese (≥30 kg/m²). PIR was divided into three groups: <1.3, 1.3-3.5, and >3.5. Race/ethnicity was categorized into five groups: Non-Hispanic White, Non-Hispanic Black, Mexican American, Other Hispanic, and Other Race. Education level was divided into three groups based on whether participants had graduated from high school: less than high school, high school, and more than high school. Marital status was classified into two groups: solitude and cohabitation.

Lifestyle behaviors included smoking and alcohol consumption. Alcohol consumption status was determined based on whether participants consumed more than 12 drinks in the past year, classifying them into drinkers and non-drinkers. Smoking status was categorized based on lifetime smoking of over 100 cigarettes and current smoking status: never (less than 100 cigarettes), former (>100 cigarettes, currently non-smoking), and current (remaining participants). Hematological indicators included serum uric acid and estimated glomerular filtration rate (eGFR). Medical comorbidities included hypertension, CVD, diabetes mellitus (DM), hyperlipidemia, and chronic kidney disease (CKD). Hypertension was defined based on previous diagnosis, use of antihypertensive medications, or measured blood pressure ≥ 140/90 mmHg. Hyperlipidemia was determined based on previous diagnosis, use of lipid-lowering medications, or a TC value of ≥240 mg/dL. DM was diagnosed based on objective testing and use of diabetes medications or insulin, with fasting glucose levels of ≥126 mg/dL or a plasma glucose level of ≥200 mg/dL at 2 hours after an oral glucose tolerance test (OGTT). Participants with abnormal glucose levels while not meeting the criteria for diabetes were classified as prediabetes. CKD was defined as an eGFR of <60 ml/min/1.73 m², corresponding to stage 3-4 kidney disease. CVD was primarily based on previous diagnoses, including myocardial infarction, angina, coronary artery disease, or heart failure.

### 2.4 Statistical analysis

Statistical analyses were conducted in strict accordance with the CDC guidelines for analyzing NHANES data, employing weighted methods to account for the complex survey design (22). Descriptive statistics were used to summarize the characteristics of the study population, with continuous variables expressed as weighted means ± standard deviations and compared using weighted linear regression, while categorical variables were presented as weighted percentages and compared using weighted chi-square tests. Multivariable linear regression and logistic regression analyses were performed to assess the impact of RC on testosterone levels and the risk of TD, respectively. Three models were included in the regression analyses, with log-transformed RC treated as a continuous variable and quartiles of RC treated as a categorical variable (Q1-Q4). Model 1 was an unadjusted model that included only the exposure variable. Model 2 was a minimally adjusted model that included the exposure variable along with age, race/ethnicity, marital status, education level, and PIR. Model 3 was the fully adjusted model, which built on Model 2 by further including BMI, smoking status, alcohol consumption, hypertension, DM, CVD, CKD, hyperlipidemia, UA, TC, HDL, and eGFR. Additionally, to visually explore the nonlinear relationships between RC and both total testosterone levels and risk of TD, smooth curve fitting and generalized additive models (GAMs) were used, adjusting for all variables included in Model 3. All linear regression results were expressed as beta coefficients with 95% confidence intervals (CI), while logistic regression results were presented as odds ratios (OR) with 95% CI.

To assess whether specific factors might influence the relationship between RC and risk of TD, subgroup analyses and interaction tests were conducted for factors including age, BMI, smoking status, DM, hypertension, CVD, and CKD. Similar to the aforementioned regression analyses, subgroup analyses included both linear and logistic regressions with log-transformed RC as a continuous variable and quartiles of RC as a categorical variable. Interaction tests across different subgroups were performed using the log-likelihood ratio test. Statistical analyses were primarily conducted using R (http://www.R-project.org, The R Foundation), supplemented by EmpowerStats software (www.empowerstats.com; X&Y solutions, Inc., Boston MA), with all statistical significance set at a two-sided p-value of <0.05.

## 3 Results

### 3.1 Baseline characteristics of the study subjects

The study included 2,848 participants, with 2,251 without TD and 597 with TD. The overall mean age was 47.73 ± 0.42 years, and the mean BMI was 28.93 ± 0.16 kg/m². Participants with TD were older (51.81 ± 0.72 vs. 46.74 ± 0.55 years, p<0.0001) and had higher BMI (33.31 ± 0.49 vs. 27.86 ± 0.16 kg/m², p<0.0001). The overall mean TC was 186.72 ± 1.07 mg/dL, with no significant difference between groups. However, those with TD had higher TG (151.84 ± 4.12 vs. 115.74 ± 2.08 mg/dL, p<0.0001) and lower HDL-C (44.17 ± 0.61 vs. 50.44 ± 0.48 mg/dL, p<0.0001). RC levels were significantly higher in the TD group (30.37 ± 0.82 vs. 23.15 ± 0.42 mg/dL, p<0.0001). Additionally, those with TD had significantly lower eGFR (90.63 ± 0.74 vs. 94.30 ± 0.57, p<0.001) and higher prevalence of hypertension (52.58% vs. 37.31%, p<0.0001), DM (32.72% vs. 13.41%, p<0.0001), hyperlipidemia (83.83% vs. 65.37%, p<0.0001), CVD (16.96% vs. 8.85%, p<0.0001), and CKD (19.87% vs. 10.28%, p<0.0001). More detailed comparison results can be found in **Table 1**.

**Table 1:** Baseline characteristics of the study population stratified by testosterone deficiency status, weighted.

Characteristics	Total participants	Participants without TD	Participants with TD	P value
Participants number	2848	2251	597	
Age, years	47.73 ± 0.42	46.74 ± 0.55	51.81 ± 0.72	< 0.0001
BMI, kg/m^2^	28.93 ± 0.16	27.86 ± 0.16	33.31 ± 0.49	< 0.0001
TC, mg/dl	186.72 ± 1.07	187.28 ± 1.12	184.43 ± 2.43	0.27
TG, mg/dl	122.80 ± 2.05	115.74 ± 2.08	151.84 ± 4.12	< 0.0001
HDL, mg/dl	49.22 ± 0.41	50.44 ± 0.48	44.17 ± 0.61	< 0.0001
LDL, mg/dl	112.94 ± 0.84	113.68 ± 0.93	109.89 ± 2.11	0.11
Non-HDL, mg/dl	137.50 ± 1.07	136.84 ± 1.13	140.26 ± 2.45	0.19
RC, mg/dl	24.57 ± 0.41	23.15 ± 0.42	30.37 ± 0.82	< 0.0001
Total testosterone, ng/dl	452.71 ± 5.15	508.79 ± 5.82	222.00 ± 3.30	< 0.0001
UA, mg/dl	6.10 ± 0.03	6.00 ± 0.03	6.53 ± 0.07	< 0.0001
eGFR	93.58 ± 0.49	94.30 ± 0.57	90.63 ± 0.74	< 0.001
Age group, %				< 0.001
20-40y	34.87	37.42	24.40	
40-60y	37.69	36.77	41.45	
≥60y	27.44	25.81	34.16	
BMI, %				< 0.0001
Normal (<25 kg/m^2^)	26.55	30.43	10.58	
Overweight (25-30 kg/m^2^)	37.98	40.07	29.37	
Obese (≥30 kg/m^2^)	35.47	29.50	60.05	
PIR, %				0.8
<1.3	20.79	20.89	20.37	
1.3-3.5	35.18	34.83	36.63	
>=3.5	44.03	44.28	43.00	
Race, %				0.53
Non-Hispanic White	70.20	69.58	72.78	
Non-Hispanic Black	8.20	8.32	7.69	
Mexican American	8.36	8.60	7.37	
Other Hispanic	6.00	6.02	5.93	
Other Race	7.23	7.48	6.23	
Education, %				0.93
Less than high school	15.78	15.63	16.41	
High school	22.57	22.62	22.36	
More than high school	61.65	61.75	61.23	
Marital status, %				0.09
Solitude	32.57	33.55	28.53	
Cohabitation	67.43	66.45	71.47	
Smoke, %				< 0.0001
Never	48.13	49.34	43.12	
Former	30.73	28.13	41.43	
Current	21.14	22.52	15.45	
Alcohol, %				< 0.001
No	21.13	18.95	30.12	
Yes	78.87	81.05	69.88	
Hypertension, %				< 0.0001
No	59.70	62.69	47.42	
Yes	40.30	37.31	52.58	
Diabetes, %				< 0.0001
No	62.78	67.98	41.38	
Prediabetes	20.04	18.61	25.91	
Yes	17.18	13.41	32.72	
Hyperlipidemia, %				< 0.0001
No	31.02	34.63	16.17	
Yes	68.98	65.37	83.83	
CVD, %				< 0.0001
No	89.57	91.15	83.04	
Yes	10.43	8.85	16.96	
CKD, %				< 0.0001
No	87.84	89.72	80.13	
Yes	12.16	10.28	19.87	

BMI, body mass index; TC, total cholesterol; TG, triglycerides; HDL-C, high-density lipoprotein cholesterol; LDL-C, low-density lipoprotein cholesterol; RC, remnant cholesterol; UA, uric acid; eGFR, estimated glomerular filtration rate; PIR, poverty income ratio; CVD, cardiovascular disease; CKD, chronic kidney disease.

Statistical methods: Continuous variables were expressed as weighted means ± standard errors and compared using weighted linear regression, while categorical variables were presented as weighted percentages and compared using weighted chi-square tests.

### 3.2 Association between remnant cholesterol and total testosterone levels and testosterone deficiency

The association between RC and total testosterone levels, as well as TD, was analyzed using three models: Model 1 (unadjusted), Model 2 (minimally adjusted), and Model 3 (fully adjusted). In Model 1, higher RC levels were significantly associated with lower total testosterone levels (β = -90.08, 95% CI: -103.68 to -76.47, p<0.0001). This association remained significant in Model 2 (β = -87.74, 95% CI: -101.22 to -74.26, p<0.0001) and Model 3 (β = -53.87, 95% CI: -77.69 to -30.06, p<0.001). Similarly, when RC was analyzed as quartiles, there was a clear trend of decreasing total testosterone levels across increasing RC quartiles (p for trend <0.001 in all models). In Model 3, participants in the highest RC quartile (Q4) had significantly lower total testosterone levels compared to those in the lowest quartile (Q1) (β = -62.19, 95% CI: -93.62 to -30.76, p<0.001).

**Figure 2:**
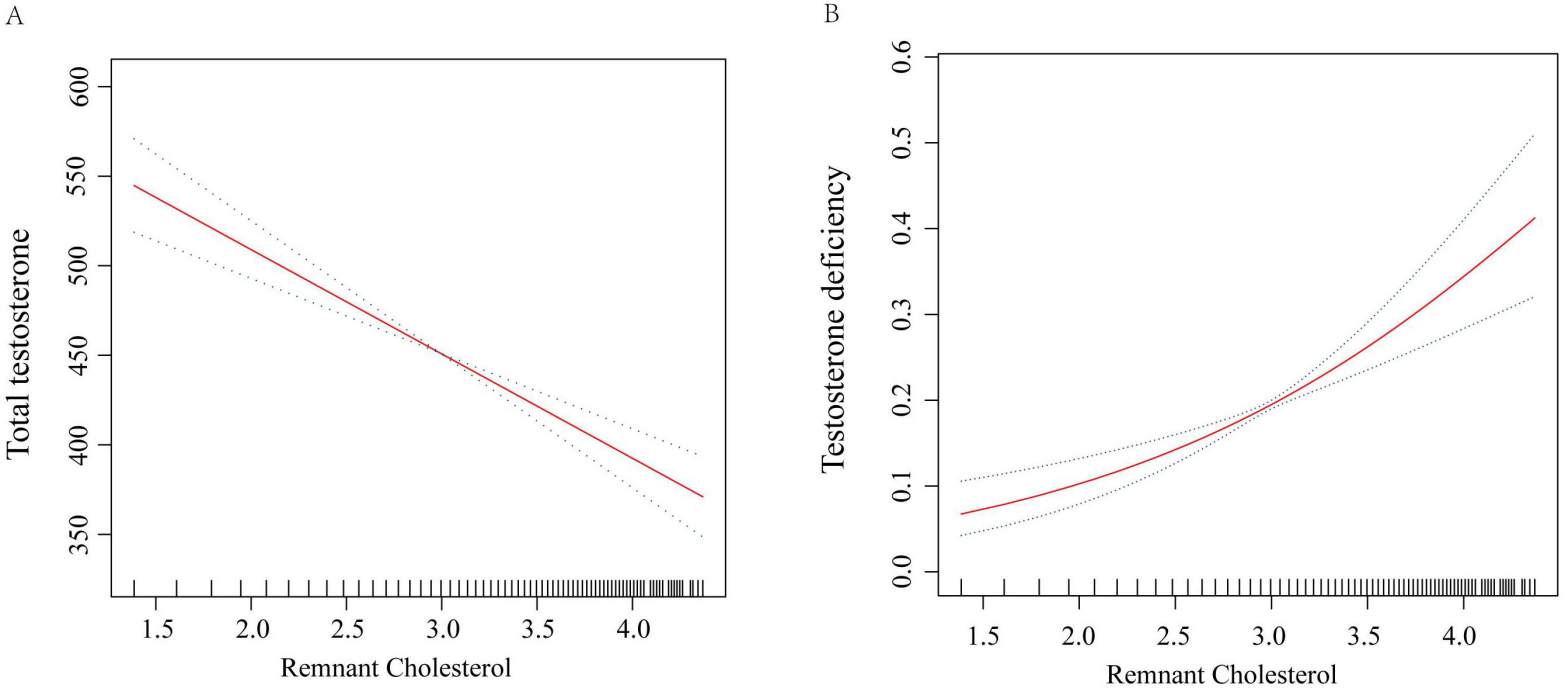
Smooth curve fit for the association between RC and testosterone levels. **(A)** Total testosterone levels; **(B)** Risk of testosterone deficiency. Adjustments were performed in Model 3, including age, race/ethnicity, marital status, education level, PIR, BMI, smoking status, alcohol consumption, hypertension, diabetes, CVD, CKD, hyperlipidemia, UA, TC, HDL-C, and eGFR. The red solid line represents ORs, and the black dashed line represents 95% CI. RC, remnant cholesterol; OR, odds ratio; CI, confidence interval; BMI, body mass index; PIR, poverty income ratio; CVD, cardiovascular disease; CKD, chronic kidney disease; UA, uric acid; TC, total cholesterol; HDL-C, high-density lipoprotein cholesterol; eGFR, estimated glomerular filtration rate.

For TD, higher RC levels were associated with an increased risk of TD. In Model 1, the OR for TD per log unit increase in RC was 2.65 (95% CI: 2.14 to 3.28, p<0.0001). This association persisted in Model 2 (OR = 2.70, 95% CI: 2.17 to 3.35, p<0.0001) and Model 3 (OR = 1.85, 95% CI: 1.29 to 2.66, p=0.002). When comparing RC quartiles, participants in Q4 had significantly higher odds of TD compared to those in Q1, with an OR of 2.15 (95% CI: 1.21 to 3.84, p=0.01) in Model 3. Detailed results can be found in **Table 2**. **Figures 2A, B** further illustrate the nonlinear relationships between RC levels and total testosterone levels as well as the risk of TD, respectively. The figures demonstrate that these associations are stable and linear.

**Table 2:** Association between remnant cholesterol and total testosterone levels and testosterone deficiency, weighted.

	Model 1	Model 2	Model 3
Total testosterone (ng/dl)-β (95%CI) p-value			
Continuous log (RC)	-90.08(-103.68, -76.47), <0.0001	-87.74(-101.22, -74.26), <0.0001	-53.87(-77.69, -30.06), <0.001
Quartile 1	Reference	Reference	Reference
Quartile 2	-18.83(-50.13, 12.47), 0.23	-18.07(-49.61, 13.46), 0.25	2.7(-27.66, 33.06), 0.86
Quartile 3	-75.96(-100.85, -51.08), <0.0001	-73.48(-97.81, -49.14), <0.0001	-36.55(-65.85, -7.25), 0.02
Quartile 4	-122.97(-143.86, -102.08), <0.0001	-120.71(-141.94, -99.48), <0.0001	-62.19(-93.62, -30.76), <0.001
P for trend	<0.0001	<0.0001	<0.001
Testosterone deficiency-OR (95% CI) p-value			
Continuous log (RC)	2.65(2.14,3.28), <0.0001	2.70(2.17,3.35), <0.0001	1.85(1.29,2.66), 0.002
Quartile 1	Reference	Reference	Reference
Quartile 2	1.62(1.07,2.43), 0.02	1.63(1.06,2.49), 0.03	1.28(0.75,2.19), 0.36
Quartile 3	2.62(1.82,3.78), <0.0001	2.67(1.84,3.87), <0.0001	1.67(1.02,2.73), 0.04
Quartile 4	4.13(2.95,5.78), <0.0001	4.22(2.96,6.01), <0.0001	2.15(1.21,3.84), 0.01
P for trend	<0.0001	<0.0001	0.005

BMI, body mass index; TC, total cholesterol; TG, triglycerides; HDL-C, high-density lipoprotein cholesterol; LDL-C, low-density lipoprotein cholesterol; RC, remnant cholesterol; UA, uric acid; eGFR, estimated glomerular filtration rate; PIR, poverty income ratio; CVD, cardiovascular disease; CKD, chronic kidney disease; DM: diabetes mellitus; CI, confidence interval; OR, odds ratio.

Model adjustments:

Model 1: Unadjusted model including only the exposure variable (RC).

Model 2: Minimally adjusted model including RC along with age, race/ethnicity, marital status, education level, and PIR.

Model 3: Fully adjusted model including variables from Model 2 plus BMI, smoking status, alcohol consumption, hypertension, DM, CVD, CKD, hyperlipidemia, UA, TC, HDL-C, and eGFR.

### 3.3 Subgroup analysis of the association between remnant cholesterol and testosterone deficiency

Subgroup analyses were conducted to examine the impact of continuous RC on total testosterone levels and TD across various demographic and health-related factors. The strongest association was observed in participants over 60 years (β = -77.27, 95% CI: -141.32 to -13.22, p=0.02; OR = 2.57, 95% CI: 1.23 to 5.37, p=0.01). Significant associations were particularly notable in never smokers (β = -60.06, 95% CI: -89.35 to -30.76, p<0.001; OR = 2.23, 95% CI: 1.35 to 3.67, p=0.003). Among participants with diabetes and CVD, the associations were not significant. However, the associations were significant in both hypertensive and non-hypertensive groups, as well as in those with and without CKD. Detailed results can be found in **Table 3**. Next, we converted continuous RC into quartiles for subgroup analysis, using Q1 as the reference. Subgroup analysis results were consistent across quartiles, showing similar trends with no significant interactions. This indicates that the relationship between RC and total testosterone as well as TD is robust across different subgroups. Detailed results can be found in **Table 4**.

**Table 3:** Subgroup analysis of the association between continuous remnant cholesterol and total testosterone levels and testosterone deficiency, weighted.

Subgroup	Total testosterone (ng/dl)-β (95%CI) p-value			Testosterone deficiency-OR (95% CI) p-value		
	β (95%CI)	P value	P for interaction	OR (95%CI)	P value	P for interaction
Age group			0.28			0.90
20-40y	-55.45(-89.96, -20.95)	0.003		1.68(0.99, 2.86)	0.06	
40-60y	-35.01(-66.14, -3.87)	0.03		1.73(1.02,2.94)	0.04	
>60y	-77.27(-141.32, -13.22)	0.02		2.57(1.23,5.37)	0.01	
BMI			0.06			0.36
Normal	-65.11(-108.22, -22.00)	0.005		2.83(1.37,5.84)	0.01	
Overweight	-65.29(-100.24, -30.35)	<0.001		2.02(1.10,3.71)	0.02	
Obese	-25.35(-62.49, 11.78)	0.17		1.41(0.87,2.28)	0.15	
Smoking status			0.97			0.47
Never	-60.06(-89.35, -30.76)	<0.001		2.23(1.35,3.67)	0.003	
Former	-43.42(-93.89, 7.05)	0.09		1.44(0.66,3.15)	0.35	
Current	-63.01(-101.94, -24.09)	0.003		2.41(1.12,5.16)	0.03	
DM			0.38			0.40
No	-49.67(-76.73, -22.62)	<0.001		1.44(1.00,2.08)	0.05	
Borderline	-82.05(-128.85, -35.26)	0.001		3.86(1.57,9.46)	0.005	
Yes	-18.55(-83.59, 46.49)	0.56		1.59(0.76,3.34)	0.21	
Hypertension			0.45			0.82
No	-43.63(-76.32, -10.95)	0.01		1.75(1.08,2.85)	0.02	
Yes	-66.7(-101.20, -32.21)	<0.001		1.98(1.10,3.56)	0.02	
CVD			0.10			0.27
No	-57.28(-81.38, -33.17)	<0.0001		1.98(1.38,2.84)	<0.001	
Yes	-18.31(-89.25, 52.63)	0.59		1.63(0.55,4.83)	0.36	
CKD			0.31			0.13
No	-45.66(-69.87, -21.46)	<0.001		1.74(1.19,2.53)	0.01	
Yes	-87.22(-138.36, -36.07)	0.002		2.67(1.37,5.21)	0.01	

RC, remnant cholesterol; CI, confidence interval; OR, odds ratio; BMI, body mass index; PIR, poverty income ratio; CVD, cardiovascular disease; CKD, chronic kidney disease; UA, uric acid; TC, total cholesterol; HDL-C, high-density lipoprotein cholesterol; eGFR, estimated glomerular filtration rate.

All analyses were adjusted for Model 3 variables, including age, race/ethnicity, marital status, education level, PIR, BMI, smoking status, alcohol consumption, hypertension, diabetes, CVD, CKD, hyperlipidemia, UA, TC, HDL-C, and eGFR, excluding the subgroup variable itself.

**Table 4:** Subgroup analysis of the association between quartiles of remnant cholesterol and total testosterone levels and testosterone deficiency, weighted.

Subgroup	Quartile 1	Quartile 2	Quartile 3	Quartile 4	P for trend	P for interaction
Total testosterone (ng/dl)-β (95%CI)						
Age group						0.56
20-40y	Reference	19.39(-18.17, 56.95)	-30.62(-65.99, 4.76)	-70(-122.94, -17.05)	0.01	
40-60y	Reference	-5.76(-51.67,40.15)	-34.43(-78.77, 9.91)	-50.28(-92.72, -7.85)	0.01	
>60y	Reference	-2.34(-58.58,53.89)	-43.53(-125.00,37.94)	-84.42(-162.79, -6.06)	0.04	
BMI						0.06
Normal	Reference	-2.03(-47.52, 43.47)	-39.93(-96.73, 16.87)	-117.02(-175.24, -58.80)	0.01	
Overweight	Reference	22.29(-23.92, 68.50)	-37.71(-85.20, 9.79)	-69.25(-117.73, -20.77)	0.001	
Obese	Reference	-10.18(-59.36, 39.00)	-21.94(-65.46, 21.58)	-20.49(-76.71, 35.74)	0.41	
Smoking status						0.60
Never	Reference	20.45(-15.67, 56.57)	-37.46(-63.83, -11.09)	-60.94(-99.02, -22.85)	<0.001	
Former	Reference	-20.74(-66.21,24.73)	-25.39(-103.29, 52.51)	-59.24(-127.90, 9.41)	0.13	
Current	Reference	-20.83(-74.78, 33.11)	-65(-121.58, -8.41)	-97.6(-146.92, -48.28)	<0.001	
DM						0.69
No	Reference	8.49(-28.58, 45.56)	-30.78(-62.44, 0.87)	-52.99(-94.37, -11.60)	0.004	
Borderline	Reference	-54.91(-110.68, 0.87)	-89.32(-137.37, 41.28)	-111.71(-178.05, -45.37)	0.002	
Yes	Reference	34.95(-40.03,109.93)	7.32(-84.44, 99.09)	-27.72(-110.75, 55.31)	0.29	
Hypertension						0.35
No	Reference	14.65(-18.71, 48.01)	-19.7(-55.47, 16.06)	-51.24(-96.38, -6.10)	0.02	
Yes	Reference	-30.78(-74.80, 13.23)	-66.06(-109.62, -22.5)	-88.86(-137.04, -40.68)	<0.001	
CVD						0.06
No	Reference	11.54(-20.50, 43.59)	-36.19(-68.18, -4.20)	-63.36(-95.72, -31.00)	<0.001	
Yes	Reference	-59.36(-154.78, 36.05)	-36.35(-130.24, 57.54)	-70.33(-187.01, 46.35)	0.32	
CKD						0.47
No	Reference	0.86(-29.52, 31.25)	-33.08(-63.15, -3.01)	-53.09(-86.20, -19.98)	<0.001	
Yes	Reference	26.24(-61.18,113.65)	-19.65(-81.74, 42.43)	-81.78(-150.79, -12.77)	0.005	
Testosterone deficiency-OR (95% CI)						
Age group						0.40
20-40y	Reference	0.82(0.30, 2.28)	1.04(0.41, 2.63)	2.10(0.89, 4.95)	0.05	
40-60y	Reference	1.24(0.45,3.38)	1.90(0.77,4.71)	1.99(0.82,4.82)	0.07	
>60y	Reference	1.75(0.78,3.94)	2.11(0.91,4.85)	2.88(0.95,8.71)	0.06	
BMI						0.28
Normal	Reference	1.75(0.74, 4.16)	2.37(0.80, 7.02)	3.24(0.77,13.55)	0.08	
Overweight	Reference	1.51(0.59,3.88)	1.24(0.49,3.11)	2.90(0.97,8.63)	0.05	
Obese	Reference	0.98(0.44,2.15)	1.59(0.78,3.23)	1.37(0.62,3.01)	0.24	
Smoking status						0.43
Never	Reference	0.89(0.43,1.85)	1.71(0.86,3.40)	2.45(1.15,5.23)	0.01	
Former	Reference	1.90(0.90,4.00)	1.88(0.81,4.34)	2.04(0.65,6.35)	0.34	
Current	Reference	1.68(0.55, 5.12)	1.49(0.54, 4.09)	3.08(0.94,10.10)	0.06	
DM						0.62
No	Reference	1.40(0.65,2.99)	1.53(0.70,3.32)	1.87(1.00,3.50)	0.05	
Borderline	Reference	2.24(0.74, 6.78)	3.86(1.54, 9.68)	5.92(1.56,22.42)	0.004	
Yes	Reference	0.71(0.29,1.72)	1.03(0.47,2.23)	1.37(0.54,3.49)	0.29	
Hypertension						0.70
No	Reference	1.50(0.76,2.93)	1.71(0.96,3.07)	2.67(1.45,4.91)	0.01	
Yes	Reference	1.08(0.55,2.16)	1.63(0.82,3.26)	1.79(0.70,4.57)	0.13	
CVD						0.07
No	Reference	1.00(0.58,1.75)	1.49(0.86,2.59)	2.17(1.23,3.84)	0.002	
Yes	Reference	4.65(1.43,15.13)	3.61(1.06,12.31)	3.32(0.68,16.26)	0.50	
CKD						0.22
No	Reference	1.28(0.68,2.42)	1.73(1.01,2.96)	2.16(1.14,4.09)	0.01	
Yes	Reference	1.28(0.52,3.17)	1.31(0.58,2.96)	2.25(0.90,5.59)	0.09	

RC, remnant cholesterol; CI, confidence interval; OR, odds ratio; BMI, body mass index; PIR, poverty income ratio; CVD, cardiovascular disease; CKD, chronic kidney disease; UA, uric acid; TC, total cholesterol; HDL-C, high-density lipoprotein cholesterol; eGFR, estimated glomerular filtration rate.

All analyses were adjusted for Model 3 variables, including age, race/ethnicity, marital status, education level, PIR, BMI, smoking status, alcohol consumption, hypertension, diabetes, CVD, CKD, hyperlipidemia, UA, TC, HDL-C, and eGFR, excluding the subgroup variable itself.

## 4 Discussion

Our study demonstrates a strong, linear association between RC and both total testosterone levels and the risk of TD, even after adjusting for various potential confounders. Subgroup analyses revealed that the association was particularly pronounced in individuals over 40 years old and non-smokers. Importantly, no significant interactions were detected across subgroups, ensuring the robustness and reliability of our results. This is the first known study to elucidate the precise relationship between RC and TD, suggesting that RC could serve as a novel biomarker for the early identification of individuals at risk for TD, potentially aiding in timely clinical interventions.

RC is a new indicator representing cholesterol content in triglyceride-rich lipoproteins (15), and it has been shown to be associated with various diseases. A study has shown a significant correlation between RC and cardiovascular mortality (23). Shi et al. found that the correlation between RC and hypertension is independent of LDL-C levels, and this relationship persists even after considering elevated TG levels (24). Another NHANES study involving 3,370 participants indicated a nonlinear positive correlation between RC and non-alcoholic fatty liver disease (25). Recently, Vargas-Vázquez et al. conducted a study on 16,113 non-diabetic American participants aged ≥20 years, showing that increased RC may raise the risk of cardiovascular disease mortality through direct atherogenic effects and indirect effects on insulin resistance (IR) (26). However, RC has little impact on cardiovascular disease mortality in individuals without systemic IR, highlighting the close relationship between RC and IR. Additionally, higher levels of RC are positively correlated with cognitive impairment, depression, and frailty in older adults (27–29). However, no studies have yet explored the relationship between RC and TD. In our study, we found a significant inverse relationship between RC levels and total testosterone levels, and a positive correlation with the risk of TD.

Currently, MetS (including obesity, dyslipidemia, hypertension, and IR) is closely associated with TD (30), and several clinical studies have shown that MetS is negatively correlated with total testosterone levels and is a risk factor for TD (31–34). IR, as a central component of MetS, is closely associated with TD (30). It reduces the secretion of testosterone by Leydig cells in the testes (35) and is independently associated with low testosterone levels (36). The underlying mechanism for the association between RC and TD is unknown and may be related to IR. Previous studies have demonstrated a close relationship between RC and IR (37–39). Research has shown that the VLDL receptor (also an RC receptor) plays a crucial role in obesity, IR, and hyperlipidemia in mice fed a high-fat refined sugar diet (HFS). Interestingly, in HFS-fed VLDL receptor knockout mice, plasma VLDL remnants increased, but these mice could not store triglycerides in adipose tissue and did not develop IR despite the HFS. Therefore, the formation of VLDL remnants due to HFS could be a precursor to obesity and IR (40–42). Previous human studies have shown that mild acute hypertriglyceridemia leads to impaired oral glucose tolerance and reduced whole-body insulin sensitivity, suggesting a potential role of TRL and its remnants (RC) in inducing IR (43). Additionally, high levels of RC can increase insulin resistance by generating lipoprotein remnants in the arteries (44, 45). RC is also associated with inflammation; it is degraded and metabolized by lipoprotein lipase, producing free fatty acids and monoacylglycerol, which trigger inflammatory responses (46). Excess remnant lipoproteins in the plasma can penetrate the arterial wall, be absorbed by macrophages and smooth muscle cells, leading to foam cell formation and low-grade inflammation, thereby raising blood glucose levels (47, 48).

In addition to RC, alcohol consumption and smoking are also potential risk factors for TD. Recent meta-analysis findings indicate that alcohol intake is associated with a significant reduction in circulating concentrations of total testosterone (mean difference [MD] = -4.02; 95% CI -6.30, -1.73) and free testosterone (MD = -0.17; 95% CI -0.23, -0.12) (49). These effects are particularly evident in healthy men exposed to chronic alcohol consumption, but not in those with a recognized diagnosis of acute alcohol intake. Mechanistically, alcohol can directly affect the hypothalamic-pituitary-gonadal axis, inhibiting the release of LH and FSH, which subsequently reduces testosterone secretion in the testes (50). The relationship between smoking and testosterone levels remains controversial. For instance, a study by Huang et al. using NHANES data showed that, compared to never smokers, current smokers had higher testosterone levels (β = 0.083, 95% CI: 0.028, 0.140) (51). However, other studies suggest that smoking can directly reduce testosterone levels and semen quality through testicular damage, with a dose-dependent relationship (52). Additionally, many mechanistic studies have confirmed that smoking can induce testicular inflammation and oxidative stress, damaging the testicular structure and consequently reducing testosterone secretion (53, 54). Therefore, the impact of smoking on testosterone levels requires further investigation through larger prospective studies to confirm these associations.

This study utilized data from NHANES, which includes a large and nationally representative sample of adult American males. This extensive dataset allows for more robust and generalizable findings. Moreover, the use of multivariable linear and logistic regression analyses, smooth curve fitting, and generalized additive models ensures that the associations between RC and testosterone levels are rigorously evaluated, accounting for various potential confounders. Understanding the relationship between RC and TD can contribute to personalized medicine approaches. For instance, individuals with high RC levels might benefit from targeted interventions to manage their cholesterol levels and prevent TD. Finally, The strong association between RC and TD underscores the importance of lipid management in public health strategies. Efforts to reduce RC levels in the population could have broader benefits, including reduced prevalence of TD and related complications.

Every study has its limitations, and ours is no exception. First, the cross-sectional nature of our study precludes establishing causal relationships, which is an inherent limitation of such designs. Future cohort studies or randomized controlled trials (RCTs) are necessary to confirm our findings. Second, the diagnosis of TD was based solely on a single measurement of testosterone levels without considering associated symptoms or signs, a limitation imposed by NHANES data. This underscores the need for future clinically based studies that incorporate a more comprehensive diagnostic approach. Third, our results are based on a U.S. population, limiting the generalizability to other populations. Validation in diverse cohorts is required to confirm these findings. Finally, NHANES lacks data on testicular volume, which prevented us from including this variable in our analyses. Future research with larger samples and better study designs is needed to confirm our results and broaden the applicability of our findings.

## 5 Conclusion

This study found a significant inverse relationship between RC levels and total testosterone levels, as well as a positive association with the risk of TD in adult American males. These findings suggest that RC could serve as a valuable biomarker for early identification of individuals at risk for TD, with implications for clinical management and public health nutrition strategies. However, our study is limited by its cross-sectional design, which precludes causal inference, and the reliance on self-reported data for some covariates. Future research should focus on longitudinal studies to confirm these associations and explore the underlying mechanisms.

## Data Availability

The datasets generated and analyzed during the current study are available from the corresponding author on reasonable request. The NHANES data are publicly available from the NCHS website (https://wwwn.cdc.gov/nchs/nhanes/Default.aspx).
